# Supplementary material for: Enhancement of Peroxydisulfate Activation for Complete Degradation of Refractory Tetracycline by 3D Self-Supported MoS2/MXene Nanocomplex
Source: Nanomaterials (Basel). 2024 Apr 30;14(9):786. doi: 10.3390/nano14090786 (PMC11085324; doi:10.3390/nano14090786)
Supplement: Supplementary file 1 [file nanomaterials-14-00786-s001.zip › nanomaterials-2989006-supplementary.pdf]

Supplementary Materials for

Enhancement of Peroxydisulfate Activation for Complete Degradation of Refractory Tetracycline by 3D Self-Supported MoS_2/MXene Nanocomplex

Yuxia Song, Runhua Chen *, Shihai Li, Shali Yu, Xiaoli Ni, Minglong Fang and Hanyun Xie

College of Life and Environmental Sciences, Central South University of Forestry and Technology, Changsha 410004, China

Correspondence: chen12@csuft.edu.cn

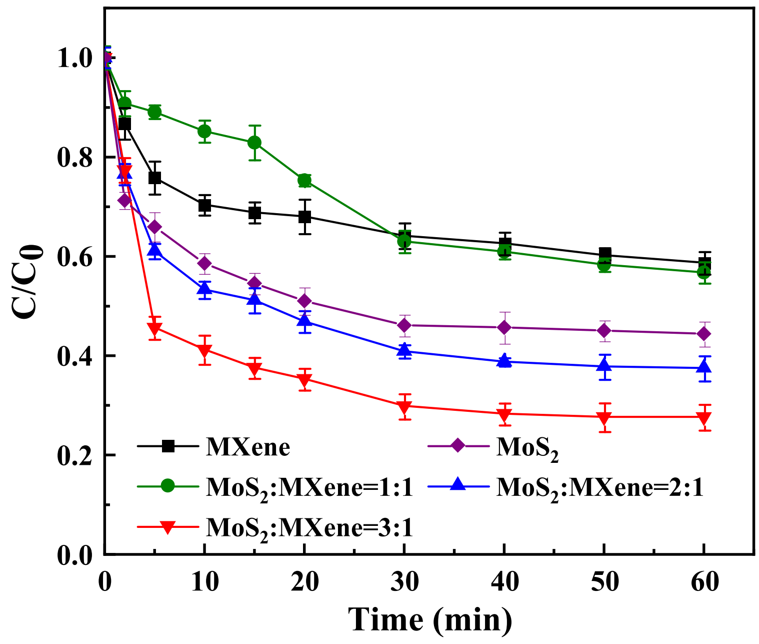

Figure S1. Effect of different materials on the degradation efficiency of TC.

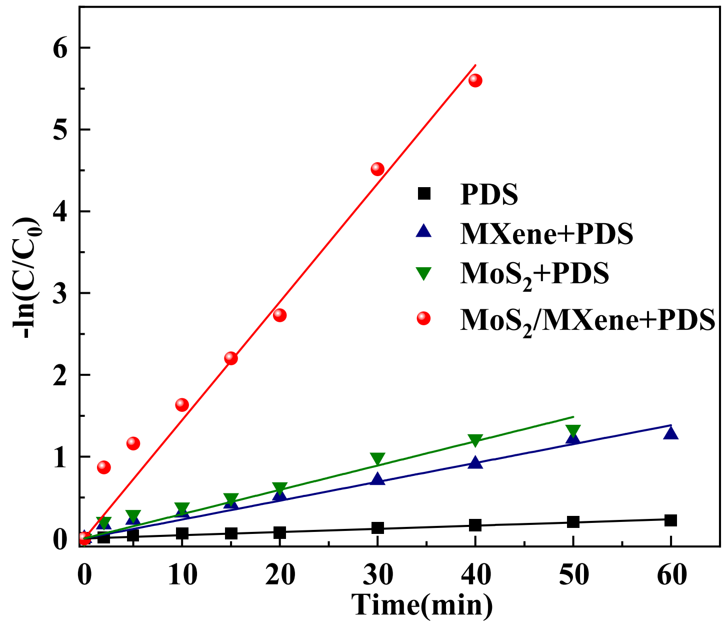

Figure S2. Kinetic fitting models for different materials/systems.

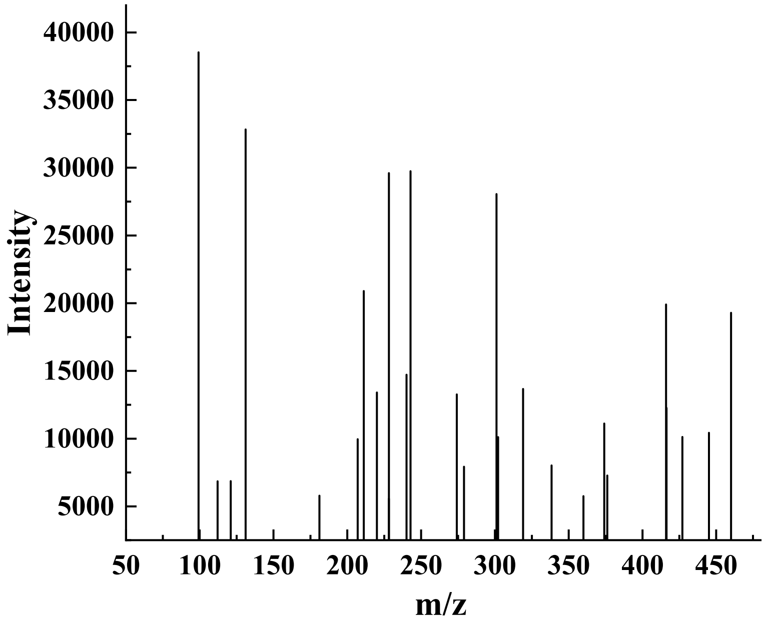

Figure S3. MS full scan spectra of intermediates in TC degradation in the MoS_2/MXene/PDS system by LC-MS.

Table S1. The elemental weight percentages and atomic percentages of MoS_2/MXene were calculated by EDX spectroscopy.

Element	Weight %	Atomic %
C	8.42	26.14
O	5.68	13.23
Mo	47.79	18.58
S	32.18	37.43
Ti	5.94	4.62

Table S2. The fitting parameters of kinetic models of TC degradation by MoS_2/MXene/PDS.

Materials/Systems	Equations	k(min $^{-1}$)	R^2
PDS	y=0.0039x	0.0039	0.992
MXene/PDS	y=0.0231x	0.0231	0.988
MoS_2/PDS	y=0.0297x	0.0297	0.983
MoS_2/MXene/PDS	y=0.1447x	0.1447	0.991

Table S3. The reaction rate constants of radicals with the corresponding scavenger.

Free Radical Species	Scavenger	Rate Constant (k) (M^{-1} s^{-1})
	Ethanol (EtOH)	(1.6-7.7) ×10^7/ (1.2-2.8) × 10^9
$SO_4^{\bullet-}$/•OH	Methanol (MeOH)	(1.6-7.7) × 10^7/9.7 × 10^8
	Tert-butanol (TBA)	(4.0-9.1) × 10^5/ (3.8 -7.6) × 10^8
$O_2^{\bullet-}$	Benzoquinone(p-BQ)	3.0 × 10^{10}

Table S4. The Elemental peak positions and contents of fresh and used MoS_2/MXene

	Fresh		Used	
Elements	Peak Positions	Contents (%)	Peak Positions	Contents (%)
C 1s	285.1eV	66.62	284.8 eV	56.32
O 1s	532.2 eV	15.77	530.9 eV	23.20
Mo 3d	228.4 eV	5.31	228.4 eV	6.85
S 2p	161.8 eV	9.29	161.9 eV	9.13
Ti 2p	456.1 eV	3.01	458.7 eV	4.49

Table S5. The group percentages of fresh and used MoS_2/MXene.

Samples	C (%)			O (%)			Mo (%)		S (%)	
	**C-C**	**C-O**	**Ti-C**	**C-O**	**Ti-O**	**C=O**	**Mo^{4+}**	**Mo^{6+}**	**S^{2-}**	**S_2^{2-}**
Fresh	32.57	5.45	1.59	16.52	5.61	5.93	5.93	0.07	15.37	0
Used	41.41	11.21	0	9.88	8.57	3.99	4.54	0.86	12.63	0.67

Table S6. Liquid-phase mass spectrometry (LC-MS) analysis of intermediates and possible structural formulae.

Number	m/z	Supposed Structure
TC	446	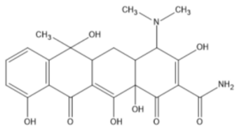
P11	460	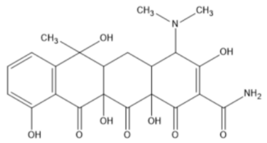
P12	374	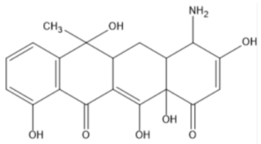
P13	302	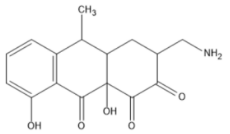
P14	242	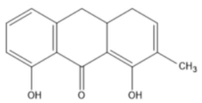
P15	240	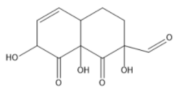
P16	220	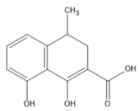

P17	228	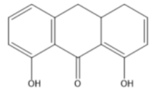
P18	99	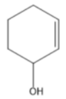
P21	416	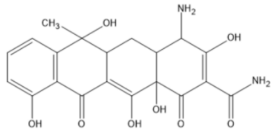
P22	376	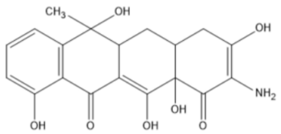
P23	338	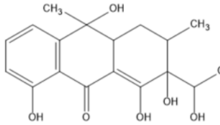
P24	360	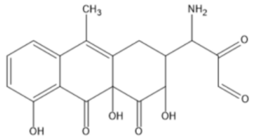
P25	211	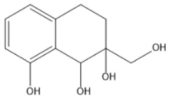
P26	121	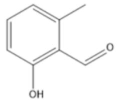
P31	427	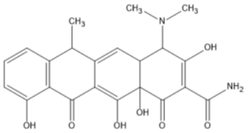
P32	319	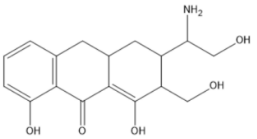
P33	279	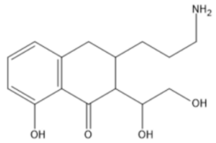
P34	207	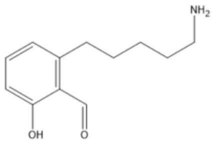

P35	181	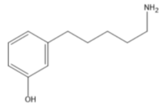
P36	301	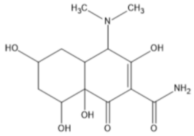
P37	274	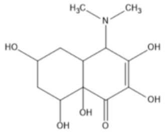
P38	131	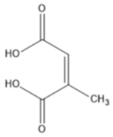
P39	112	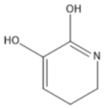
